# Association of blood urea nitrogen with all-cause and cardiovascular mortality in hyperlipidemia: NHANES 1999–2018

**DOI:** 10.1186/s12944-024-02158-1

**Published:** 2024-06-03

**Authors:** Jing Shen, Zhen Wang, Yong Liu, Tao Wang, Xiao-Yu Wang, Xin-Hui Qu, Zhi-Ping Chen, Xiao-Jian Han

**Affiliations:** 1 Institute of Geriatrics, Jiangxi Provincial People’s Hospital, The First Affiliated Hospital of Nanchang Medical College, 152 Aiguo Road, Nanchang, Jiangxi 330006 P.R. China; 2 School of Public Health, Nanchang University, Nanchang, Jiangxi 330029 China; 3 The Second Department of Neurology, Jiangxi Provincial People’s Hospital, The First Affiliated Hospital of Nanchang Medical College, 152 Aiguo Road, Nanchang, Jiangxi 330006 P.R. China

**Keywords:** Blood urea nitrogen, Patients with hyperlipidemia, NHANES, Mortality

## Abstract

**Objective:**

Although blood urea nitrogen (BUN) has a crucial impact on many diseases, its effect on outcomes in patients with hyperlipidemia remains unknown. The study aimed to investigate the relationships between BUN levels and all-cause and cardiovascular disease (CVD) mortality in individuals with hyperlipidemia.

**Methods:**

This analysis comprised 28,122 subjects with hyperlipidemia from the National Health and Nutrition Examination Survey (NHANES) spanning 1999 to 2018. The risk of BUN on mortality was evaluated using weighted Cox regression models. Additionally, to illustrate the dose-response association, the restricted cubic spline (RCS) was used.

**Results:**

During the observation period, 4276 participant deaths were recorded, of which 1206 were due to CVD. Compared to patients with hyperlipidemia in the third BUN quintile, the hazard ratios (HRs) for all-cause mortality were 1.26 (95% CIs: 1.09, 1.45) and 1.22 (95% CIs: 1.09, 1.37) for patients in the first and fifth quintiles of BUN, respectively. The HRs for CVD mortality among patients in the fifth quintile of BUN were 1.48 (95% CIs: 1.14, 1.93). BUN levels were found to have a U-shaped association with all-cause mortality and a linear association with CVD mortality using restricted triple spline analysis.

**Conclusions:**

This study revealed that both low and high BUN levels in patients with hyperlipidemia are associated with heightened all-cause mortality. Furthermore, elevated BUN levels are also associated with increased CVD mortality. The findings indicate that patients with hyperlipidemia may face an elevated risk of death if they have abnormal BUN levels.

**Supplementary Information:**

The online version contains supplementary material available at 10.1186/s12944-024-02158-1.

## Introduction

The term hyperlipidemia refers to the dysregulation of lipid metabolism, mainly caused by genetics, obesity, hypertension, diabetes, and an unhealthy lifestyle [1]. Between 2017 and 2020, approximately 86 million American adults aged 20 or older had total cholesterol levels exceeding 200 mg/dL, as reported by the American Heart Association [2]. Dyslipidemia is a significant adverse factor in the prognosis of various diseases. More than 3.8 million people die of CVD each year in Europe [3]. It highlights the urgent need for a thorough understanding and exploration of hyperlipidemia risk factors to reduce mortality.

Urea, a principal nitrogenous end product derived from the catabolism of proteins and amino acids, is predominantly excreted through renal pathways [4]. In clinical practice, BUN concentration is commonly used to reflect the amount of nitrogen coming from urea in the blood [5]. As indicated by previous studies, BUN has also been linked to disease progression, such as stroke, diabetes, and chronic liver disease [6–8]. Research indicates that dyslipidemia contributes to insulin resistance and endothelial cell inflammation [9, 10]. Additionally, BUN levels are linked with inflammation and vascular complications in individuals with chronic kidney disease (CKD), thereby heightening the incidence and mortality of CKD [11]. Despite this, the correlation between the risk of death and BUN levels in hyperlipidemic patients remains unknown.

Based on the these studies, this study hypothesizes that in patients with hyperlipidemia, elevated BUN levels may increase the risk of mortality. Using data from NHANES, the study explored the correlations between all-cause and CVD mortality and BUN levels among adult patients with hyperlipidemia. A U-shaped correlation was found between BUN and all-cause mortality, and a linear correlation was observed with CVD mortality in patients with hyperlipidemia.

## Methods

### Study population

**Fig. 1:**
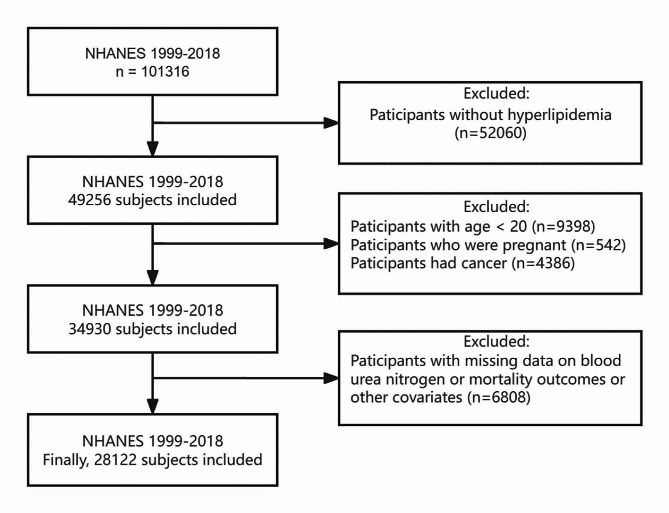
Research flow chart

The NHANES is an ongoing project of the National Center for Health Statistics (NCHS) [12]. The database comprises information obtained from interviews, mobile vehicle inspections, and laboratory tests, collected adopting multi-stage probability sampling method. NHANES was approved by the Research Ethics Review Board of NCHS, and each participant has provided signed informed consent. This study utilized data from ten cycles of the NHANES spanning the years 1999 to 2018. These data are freely available on the Centers for Disease Control and Prevention (CDC) website [13]. During this period, 101,316 individuals participated, including 49,256 patients with hyperlipidemia. The definition of hyperlipidemia includes total cholesterol of ≥ 200 mg/dL, triglycerides of ≥ 150 mg/dL, low-density lipoprotein of ≥ 130 mg/dL, or high-density lipoprotein < 50 mg/dL for females and < 40 mg/dL for males, or taking cholesterol-lowering medications [14]. After excluding individuals under 20 years of age (*n* = 9398), pregnant individuals (*n* = 542), those with cancer (*n* = 4386) at baseline, as well as those with missing data or lacking mortality follow-up (*n* = 6808), the final sample included 28,122 individuals (Fig. 1).

### Exposure measurement

The BUN level was quantitatively determined using the enzymatic conductivity rate method. The measurements were conducted with the Hitachi Model 917 multichannel analyzer form 1999 to 2000, Beckman Synchron LX20 from 2001 to 2007, Beckman UniCel® DxC800 Synchron from 2008 to 2016 and Roche Cobas 6000 from 2017 to 2018 [15–18].

### Ascertainment of mortality

Mortality statistics were obtained from the publicly accessible Linked Mortality File (LMF) provided by the CDC, and the deadline for death follow-up was December 31, 2019 [19]. The definition of CVD deaths adhered to the 10th revision of the International Statistical Classification of Diseases, Injuries, and Causes of Death (ICD-10) guidelines.

### Covariates

Data regarding age, race/ethnicity, gender, education level, physical activity, household income-to-poverty ratios (PIR), smoking status, and history of CVD were gathered using a standardized questionnaire. Race/ethnicity was divided into Mexican American, non-Hispanic Black, non-Hispanic White, and other [20]. Education level was classified as college or higher, high school, and less than high school. PIR was divided into three groups: PIR ≤ 1.30, PIR ranging from 1.31 to 3.50, and PIR above 3.50 [21]. There were three categories for smoking status: former smokers, current smokers, and never smokers, based on participants’ answers [21]. For physical activity, individuals were classified as active or inactive based on whether they engaged in more than 10 min of moderate or vigorous physical exercise during their leisure time each week [22]. CVD includes self-reported stroke, congestive angina, coronary heart disease, heart attack, or heart failure [23]. Protein intake and alcohol consumption were assessed through one or two 24-hour diet surveys. Alcohol consumption was divided into heavy drinking, moderate drinking, and none. None was termed as consuming of 0 g/day of alcohol. Drinking between 0.1 and 13.9 g/day for women and 0.1–27.9 g/day for men is considered moderate. Heavy drinking was termed as alcohol consumption of ≥ 28 g/day for males and ≥ 14 g/day for females [21]. Systolic blood pressure (SBP), diastolic blood pressure (DBP), height, and weight were measured in a mobile examination van. Body mass index (BMI) is sorted as ≥ 30 kg/m^2^, < 25 kg/m^2^, and 25 to 29.9 kg/m^2^ [24]. Hypertension was defined as self-reported hypertension, taking antihypertensive medication, or DBP ≥ 80 mmHg and/or SBP ≥ 130 mmHg [25]. Diabetes was described as medically diagnosed, fasting blood glucose level of ≥ 126 mg/dL, or glycosylated hemoglobin of ≥ 6.5% [26]. The equation developed by the CKD Epidemiology Collaboration was used to estimate the glomerular filtration rate (eGFR) [27]. Additionally, antihyperlipidemic medication, aspartate aminotransferase (AST), albumin, serum uric acid, and alanine aminotransferase (ALT) were included according to previous studies [22, 28].

### Statistical analysis

Following the NHANES analysis guidelines, all statistical analyses took into consideration the stratified survey design factors. To better investigate the effect of BUN on mortality, patients were categorized into five groups based on quintiles of BUN. Categorical variables were expressed as frequencies (%) and continuous variables as means (SD) in the analysis of baseline characteristics. The chi-square test and one-way ANOVA were used to detect group differences for categorical and continuous variables. By examining the Schoenfeld residuals, the study assessed the proportional hazards assumption and found no notable violations of this assumption. The relation of BUN levels to mortality was analyzed using Cox regression models. Three models were developed in this study. Model 1 included adjustments for race/ethnicity, age, and sex. Model 2 expanded upon Model 1 by adjusting for PIR, education level, BMI, alcohol intake, smoking status, protein intake, and physical activity. Finally, adjustments were made to Model 3 based on Model 2, including adjustments for antihyperlipidemic medications, CVD, diabetes, hypertension, eGFR, albumin, ALT, AST, and serum uric acid.

Stratified analysis was conducted for this study, and the factors included in the stratification were sex, race, age, PIR, education, physical activity, alcohol intake, smoking status, eGFR, BMI, CVD, hypertension, diabetes, and medications. Potential interactions between the aforementioned stratification factors and BUN were investigated. Subsequently, the observed associations between BUN levels and mortality rates related to both CVD and all-cause were visualized using the RCS method.

Sensitivity analysis was carried out to evaluate the robustness of the results. First, to mitigate the potential bias from reverse causality, individuals who passed away within two years prior to follow-up were excluded. Secondly, individuals with CVD at the start were excluded. All analyses were performed using R 4.2.3, with a two-sided *P*-value of < 0.05 considered statistically significant.

## Results

### Baseline characteristics of participants

Among 28,122 adult hyperlipidemia participants (mean age 48.52 ± 15.85 years, 48.88% men), the weighted mean BUN level was 4.89 mmol/L. Participants with higher BUN levels tended to be older, male, hypertensive, diabetic, have cardiovascular issues, consume higher levels of protein, take lipid-lowering medications, exhibit higher serum uric acid concentrations, and were less likely to be current smokers or have a normal weight (Table 1).

**Table 1:** Baseline characteristics of patients with hyperlipidemia

		BUN, mmol/L					
Characteristic	Overall	Q1 < 3.60	Q2 3.60-4.29	Q3 4.30-5.00	Q4 5.01-6.09	Q5 > 6.09	*P* value
**Participants**	28,122	7712	5111	4954	4877	5468	
**Age, years**	48.52 (15.85)	41.99 (13.88)	44.87 (14.60)	47.99 (15.03)	51.62 (14.91)	59.21 (15.21)	< 0.001
**Sex**							< 0.001
Female	14,484 (51.12)	4921 (64.19)	2754 (54.01)	2337 (47.89)	2142 (41.91)	2330 (41.79)	
Male	13,638 (48.88)	2791 (35.81)	2357 (45.99)	2617 (52.11)	2735 (58.09)	3138 (58.21)	
**Race/ethnicity**							< 0.001
Mexican American	5307 (8.22)	1455 (9.67)	1097 (9.69)	970 (8.19)	897 (7.05)	888 (5.81)	
Non-Hispanic White	12,729 (70.02)	3085 (63.09)	2084 (66.99)	2225 (70.43)	2412 (75.16)	2923 (77.52)	
Non-Hispanic Black	5374 (9.82)	1932 (14.88)	1009 (10.37)	875 (8.35)	722 (6.38)	836 (6.91)	
Others	4712 (11.94)	1240 (12.35)	921 (12.94)	884 (13.03)	846 (11.42)	821 (9.76)	
**Education level**							< 0.001
Less than high school	7746 (17.39)	2096 (19.41)	1370 (16.84)	1304 (15.59)	1273 (15.26)	1703 (19.01)	
High school	6717 (25.33)	1900 (26.34)	1168 (24.01)	1165 (24.87)	1143 (25.16)	1341 (25.84)	
College or higher	13,659 (57.29)	3716 (54.25)	2573 (59.15)	2485 (59.54)	2461 (59.58)	2424 (55.15)	
**PIR**							< 0.001
≤ 1.30	8760 (21.23)	2874 (28.22)	1607 (21.34)	1440 (19.72)	1308 (16.09)	1531 (17.71)	
1.31–3.50	10,685 (35.88)	2863 (36.91)	1921 (35.98)	1840 (34.26)	1810 (34.43)	2251 (37.43)	
> 3.50	8677 (42.89)	1975 (34.86)	1583 (42.68)	1674 (46.02)	1759 (49.48)	1686 (44.86)	
**BMI, kg/m** ^**2**^							< 0.001
< 25.0	6529 (23.89)	1937 (27.16)	1181 (23.91)	1113 (23.60)	1090 (21.69)	1208 (21.66)	
25.0-29.9	9915 (35.09)	2469 (31.16)	1769 (33.97)	1796 (36.55)	1883 (39.17)	1998 (36.27)	
≥ 30.0	11,678 (41.02)	3306 (41.68)	2161 (42.12)	2045 (39.85)	1904 (39.14)	2262 (42.08)	
**Smoking status**							< 0.001
Never	14,911 (52.53)	3990 (48.79)	2823 (55.46)	2687 (53.41)	2601 (53.17)	2810 (53.46)	
Former	7276 (25.67)	1381 (18.41)	1165 (22.12)	1311 (27.24)	1439 (29.43)	1980 (34.44)	
Current	5935 (21.80)	2341 (32.80)	1123 (22.42)	956 (19.35)	837 (17.41)	678 (12.10)	
**Alcohol intake**							< 0.001
None	20,485 (68.55)	5682 (70.26)	3697 (68.25)	3512 (66.91)	3449 (65.53)	4145 (71.13)	
Moderate drinking	4143 (16.15)	970 (13.17)	754 (15.85)	801 (17.31)	811 (18.74)	807 (16.99)	
Heavy drinking	3494 (15.30)	1060 (16.58)	660 (15.90)	641 (15.78)	617 (15.72)	516 (11.87)	
**Physical activity**							< 0.001
Inactive	13,897 (42.60)	3765 (43.58)	2440 (41.41)	2334 (40.84)	2375 (41.07)	2983 (45.79)	
Active	14,225 (57.40)	3947 (56.42)	2671 (58.59)	2620 (59.16)	2502 (58.93)	2485 (54.21)	
**Diabetes**	5319 (13.90)	1050 (10.48)	784 (11.09)	817 (11.19)	956 (14.49)	1712 (23.99)	< 0.001
**Hypertension**	16,483 (54.41)	3693 (46.35)	2699 (48.40)	2924 (53.48)	3033 (57.32)	4134 (70.33)	< 0.001
**CVD**	3358 (9.46)	540 (5.91)	409 (6.46)	435 (6.72)	630 (10.58)	1344 (19.40)	< 0.001
**Medications**	11,673 (38.39)	2243 (28.53)	1686 (30.11)	1914 (35.17)	2235 (42.55)	3595 (60.32)	< 0.001
**Protein intake, g**	82.37 (37.42)	72.90 (34.02)	80.27 (36.06)	85.28 (37.35)	89.65 (37.68)	87.88 (40.20)	< 0.001
**eGFR, mL/min/1.73m** ^**2**^	103.96 (24.19)	117.19 (20.47)	110.15 (20.39)	105.18 (19.87)	97.87 (19.85)	83.25 (25.14)	< 0.001
**Albumin, g/L**	42.74 (3.32)	42.21 (3.51)	42.84 (3.22)	43.15 (3.14)	43.02 (3.15)	42.69 (3.37)	< 0.001
**ALT, U/L**	26.91 (25.89)	26.39 (20.87)	27.73 (22.01)	26.99 (22.05)	27.33 (23)	26.31 (39.24)	< 0.001
**AST, U/L**	25.57 (17.29)	26.07 (18.97)	25.87 (19.94)	24.98 (16.10)	25.50 (17.11)	25.19 (12.43)	< 0.001
**Serum uric acid, umol/L**	329.30 (84.84)	305.74 (77.88)	322.46 (81.02)	329.64 (80.46)	338.34 (82.63)	361.24 (93.11)	< 0.001

Values are weighted mean (SD) for continuous variables or numbers (weighted %) for categorical variables. BUN, blood urea nitrogen; PIR, poverty income ratio; BMI, body mass index; CVD, cardiovascular disease; ALT, alanine aminotransferase; AST, aspartate aminotransferase

### Associations between BUN and mortality

**Fig. 2:**
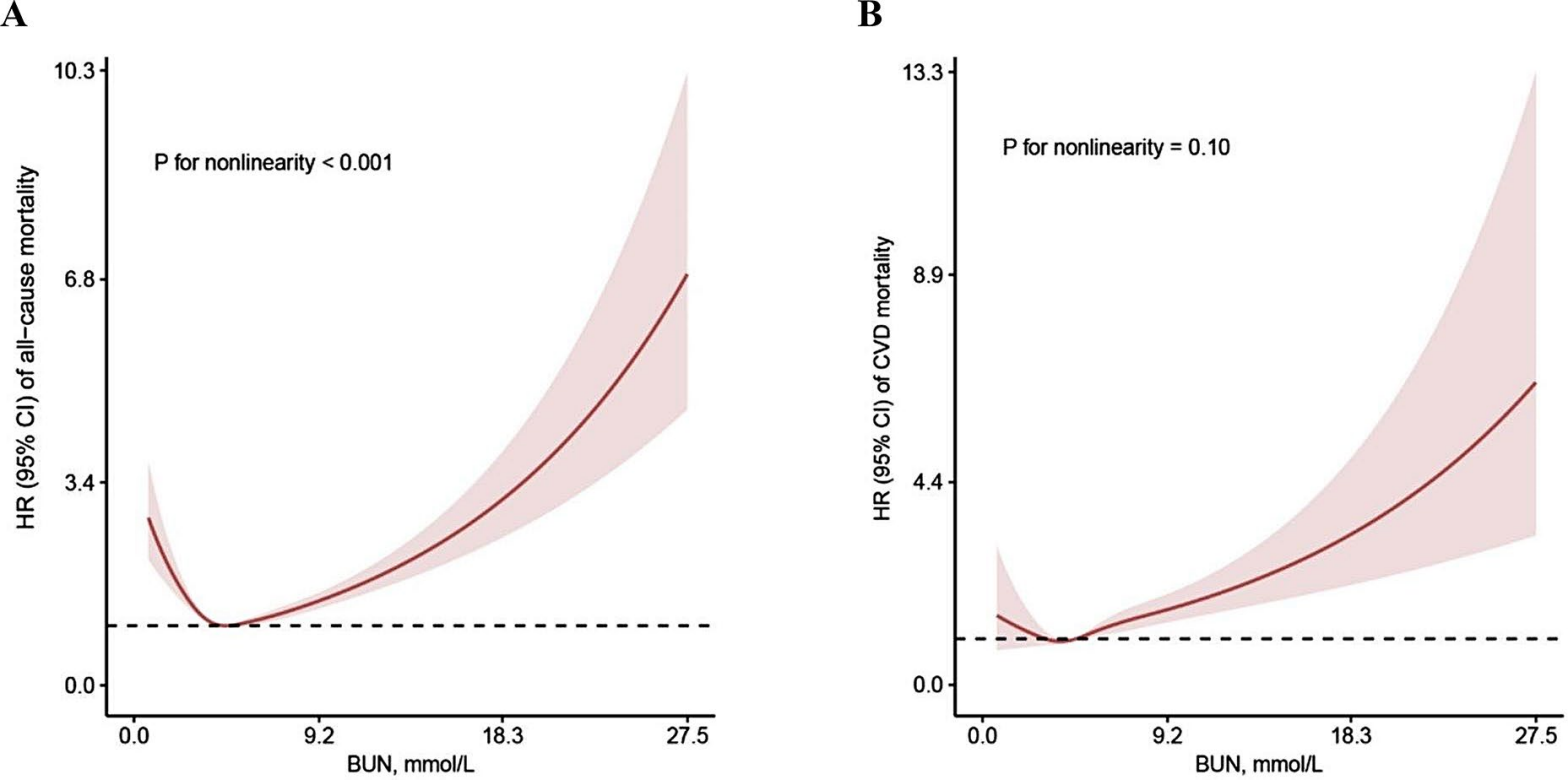
The multivariable adjusted restricted cubic splines for associations of BUN levels with all-cause (A) and CVD (B) mortality from NHANSE 1999–2018

It was observed that there is a significant association between lower and higher BUN levels and an elevated risk of all-cause mortality after multivariable adjustment. Additionally, a strong correlation was found between elevated BUN levels and a higher risk of CVD mortality (Table 2). Multivariable-adjusted HRs (95% CI) for quintiles of BUN levels were 1.26 (1.09, 1.45), 1.08 (0.93, 1.25), 1(ref), 1.02 (0.89, 1.17), and 1.22 (1.09, 1.37) (*P* for trend < 0.001), respectively. Furthermore, it was 1.03 (0.74, 1.43), 1.24 (0.91, 1.69), 1(ref), 1.27 (0.97, 1.65), and 1.48 (1.14, 1.93) (*P* for trend = 0.042) for CVD mortality. BUN levels and all-cause mortality exhibited a U-shaped association (*P* < 0.001 for nonlinearity) in the RCS, while CVD mortality demonstrated a linear relationship (*P* = 0.10 for nonlinearity) (Fig. 2).

### Stratified and sensitivity analyses

A significant interaction was observed among PIR, alcohol intake, medications, and BUN levels in relation to all-cause mortality in the stratified analyses (*P* < 0.05) (Table 2). When compared to the reference group (Q3), subgroups with a PIR ≤ 1.30 exhibited associations between low (Q1) and high (Q4 and Q5) levels of BUN and all-cause mortality, with HRs (95% CIs) of 1.52 (1.22, 1.90), 1.24 (1.01, 1.52), and 1.31 (1.03, 1.67), respectively. Among subgroups with a PIR > 3.50, high BUN levels (Q5) were linked to all-cause mortality, with HRs (95% CI) of 1.41 (1.11, 1.80). Similar trends were observed in the subgroups of alcohol intake and medications. However, no statistically significant interaction was detected between the concentration of BUN and the stratification variables regarding CVD mortality in the study (Supplementary Table 1).

**Table 2:** Association of blood urea nitrogen with all-cause and CVD mortality in patients with hyperlipidemia

	BUN, mmol/L					
	Q1 < 3.60	Q2 3.60-4.29	Q3 4.30-5.00	Q4 5.01-6.09	Q5 > 6.09	*P* trend
All-cause mortality						
Model1	1.55 (1.34, 1.78)	1.17 (1.01, 1.34)	1(ref)	1.08 (0.95, 1.22)	1.50 (1.34, 1.67)	< 0.001
Model2	1.29 (1.12, 1.48)	1.11 (0.96, 1.28)	1(ref)	1.12 (0.99, 1.26)	1.50 (1.34, 1.68)	< 0.001
Model3	1.26 (1.09, 1.45)	1.08 (0.93, 1.25)	1(ref)	1.02 (0.89, 1.17)	1.22 (1.09, 1.37)	< 0.001
**CVD mortality**						
Model1	1.25 (0.89, 1.75)	1.34 (0.97, 1.85)	1(ref)	1.37 (1.05, 1.79)	2.01 (1.57, 2.57)	< 0.001
Model2	1.03 (0.74, 1.45)	1.26 (0.91, 1.74)	1(ref)	1.42 (1.09, 1.87)	1.98 (1.53, 2.55)	< 0.001
Model3	1.03 (0.74, 1.43)	1.24 (0.91, 1.69)	1(ref)	1.27 (0.97, 1.65)	1.48 (1.14, 1.93)	0.042

HR (95% CI) was estimated by weighted Cox regression analysis. Model 1: adjusted for age, sex, race/ethnicity. Model 2: Model 1 + education, PIR, smoking status, alcohol intake, protein intake, physical activity, BMI. Model 3: Model 2 + diabetes, hypertension, CVD, medications, eGFR, albumin, ALT, AST, serum uric acid

**Table 3:** Subgroup analysis of the association between BUN levels and all-cause mortality in patients with hyperlipidemia

	BUN, mmol/L					
Subgroups	Q1 < 3.60	Q2 3.60-4.29	Q3 4.30-5.00	Q4 5.01-6.09	Q5 > 6.09	*P* interaction
**Age, years**						0.072
< 60	1.24 (0.99, 1.55)	1.20 (0.95, 1.53)	1(ref)	1.17 (0.90, 1.53)	1.48 (1.15, 1.91)	
≥ 60	1.03 (0.85, 1.25)	0.92 (0.77, 1.09)	1(ref)	0.97 (0.82, 1.13)	1.15 (1.02, 1.30)	
**Sex**						0.757
Female	1.26 (1.04, 1.54)	1.14 (0.92, 1.41)	1(ref)	1.07 (0.88, 1.30)	1.26 (1.06, 1.49)	
Male	1.28 (1.05, 1.55)	1.02 (0.83, 1.25)	1(ref)	0.99 (0.83, 1.20)	1.22 (1.04, 1.42)	
**Race/ethnicity**						0.056
Non-Hispanic White	1.22 (1.02, 1.47)	0.99 (0.82, 1.19)	1(ref)	1.00 (0.86, 1.17)	1.18 (1.03, 1.35)	
Other	1.38 (1.09, 1.75)	1.38 (1.11, 1.72)	1(ref)	1.08 (0.87, 1.35)	1.44 (1.21, 1.73)	
**Education level**						0.479
Less than high school	1.16 (0.94, 1.44)	1.16 (0.90, 1.49)	1(ref)	1.15 (0.94, 1.42)	1.33 (1.11, 1.59)	
High school	1.38 (1.03, 1.84)	1.07 (0.80, 1.41)	1(ref)	0.83 (0.64, 1.08)	1.08 (0.88, 1.31)	
College or higher	1.20 (0.97, 1.49)	1.03 (0.83, 1.28)	1(ref)	1.08 (0.87, 1.34)	1.22 (0.99, 1.50)	
**PIR**						0.031
≤ 1.30	1.52 (1.22, 1.90)	1.19 (0.92, 1.52)	1(ref)	1.24 (1.01, 1.52)	1.31 (1.03, 1.67)	
1.31–3.50	1.05 (0.84, 1.30)	1.00 (0.81, 1.24)	1(ref)	0.82 (0.67, 0.99)	1.03 (0.88, 1.21)	
> 3.50	1.26 (0.97 1.63)	1.04 (0.75, 1.46)	1(ref)	1.15 (0.88, 1.51)	1.41 (1.11, 1.80)	
**BMI, kg/m** ^**2**^						0.089
< 25.0	1.60 (1.21, 2.10)	0.91 (0.68, 1.21)	1(ref)	1.16 (0.91, 1.47)	1.20 (0.93, 1.54)	
25.0-29.9	1.15 (0.89, 1.49)	1.00 (0.78, 1.28)	1(ref)	0.98 (0.77, 1.24)	1.14 (0.91, 1.43)	
≥ 30.0	1.13 (0.90, 1.42)	1.25 (1.00, 1.56)	1(ref)	1.00 (0.81, 1.23)	1.33 (1.12, 1.59)	
**Smoking status**						0.640
Never	1.10 (0.88, 1.37)	0.96 (0.78, 1.20)	1(ref)	0.97 (0.79, 1.20)	1.16 (0.96, 1.41)	
Former	1.25 (0.97, 1.61)	1.14 (0.87, 1.49)	1(ref)	1.01 (0.80, 1.28)	1.15 (0.93, 1.41)	
Current	1.40 (1.08, 1.81)	1.19 (0.87, 1.61)	1(ref)	1.17 (0.84, 1.63)	1.46 (1.08, 1.88)	
**Alcohol intake**						0.008
None	1.15 (0.97, 1.38)	1.04 (0.88, 1.24)	1(ref)	1.04 (0.89, 1.21)	1.21 (1.06, 1.39)	
Moderate drinking	1.07 (0.76, 1.50)	1.25 (0.88, 1.76)	1(ref)	0.74 (0.51, 1.06)	1.00 (0.75, 1.33)	
Heavy drinking	1.98 (1.29, 3.02)	1.22 (0.71, 2.08)	1(ref)	1.18 (0.71, 1.94)	1.54 (1.04, 2.26)	
**Physical activity**						0.068
Inactive	1.41 (1.19, 1.67)	1.05 (0.89, 1.25)	1(ref)	0.96 (0.82, 1.13)	1.23 (1.06, 1.43)	
Active	1.06 (0.84, 1.34)	1.11 (0.88, 1.40)	1(ref)	1.07 (0.88, 1.30)	1.18 (1.01, 1.37)	
**Diabetes**						0.271
Yes	1.13 (0.85, 1.50)	1.19 (0.91, 1.56)	1(ref)	1.24 (0.94, 1.62)	1.45 (1.13, 1.86)	
No	1.29 (1.08, 1.53)	1.05 (0.87, 1.26)	1(ref)	0.97 (0.83, 1.12)	1.14 (1.00, 1.30)	
**Hypertension**						0.175
Yes	1.20 (1.03, 1.40)	1.15 (0.98, 1.34)	1(ref)	0.99 (0.84, 1.16)	1.21 (1.06, 1.37)	
No	1.35 (1.03 1.77)	0.91 (0.66, 1.24)	1(ref)	1.09 (0.84, 1.43)	1.25 (0.96, 1.63)	
**CVD**						0.466
Yes	1.19 (0.88, 1.60)	0.95 (0.69, 1.30)	1(ref)	1.11 (0.86, 1.43)	1.25 (1.00, 1.56)	
No	1.28 (1.07, 1.53)	1.13 (0.96, 1.34)	1(ref)	0.99 (0.84, 1.17)	1.22 (1.06, 1.40)	
**Medications**						
Yes	1.22 (0.99, 1.50)	1.01 (0.83, 1.23)	1(ref)	1.02 (0.86, 1.22)	1.29 (1.10, 1.51)	0.034
No	1.32 (1.09, 1.61)	1.17 (0.95, 1.44)	1(ref)	1.05 (0.84, 1.30)	1.08 (0.89, 1.30)	
**eGFR, mL/min/1.73m** ^**2**^						
< 90	1.18 (0.88, 1.57)	1.07 (0.88, 1.29)	1(ref)	1.05 (0.89, 1.25)	1.41 (1.22, 1.62)	0.059
≥ 90	1.24 (1.08, 1.43)	1.10 (0.91, 1.43)	1(ref)	1.09 (0.91, 1.31)	1.14 (0.95, 1.37)	

Values are weighted hazard ratio (95% confidence interval). Models adjusted for age, sex, race/ethnicity, education level, PIR, BMI, smoking status, alcohol intake, protein intake, physical activity, diabetes, hypertension, CVD, medications, eGFR, Albumin, ALT, AST, serum uric acid, excluding the stratifying variable

Consistent results were obtained in sensitivity analyses that removed participants who died within 2 years of the start of the follow-up (Supplementary Table 2). Furthermore, after excluding patients who had CVD at baseline, the link of BUN levels and CVD mortality was weakened (Supplementary Table 3).

## Discussion

This research is the first inverstigation of the connection between BUN levels and mortality in individuals with hyperlipidemia. In this extensive and prospective investigation involving a cohort of American individuals with hyperlipidemia, a U-shaped correlation between BUN levels and all-cause mortality, as well as a linear correlation with CVD mortality, were demonstrated. These associations remained significant after adjusting for various factors, including fundamental characteristics, behaviors, health history, and the use of lipid-lowering medications at baseline.

Epidemiological research has indicated a relationship between BUN and disease progression, as well as an elevated risk of mortality, but the findings have been inconsistent. In the general population, a research conducted using data from NHANES spanning the years 1999 to 2006 revealed that participants with elevated BUN levels, compared to those in the first quartile of BUN levels, exhibited an elevated risk of dying from CVD and all-cause [28]. However, data from the UK Biobank and Health Screening Survey X69 showed that BUN levels and mortality displayed a U-shaped relationship [29]. Various similar findings have been observed in clinical studies. A study conducted on 459 Japanese patients diagnosed with chronic kidney disease observed that a significant indicator for the development of renal disease is elevated BUN concentrations [30]. In individuals with chronic liver disease, a U-shaped correlation was observed between the development of liver fibrosis or dysfunction and urea levels [8]. These discrepancies in results may be attributed to variations in sample sizes, clinical features, or adjustments for confounding variables. Nevertheless, evidence is still lacking regarding the potential effect of BUN in patients with hyperlipidemia, particularly in relation to mortality outcomes. The results of this study on the connection between all-cause mortality and BUN align with those mentioned above, conducted in the UK Biobank, indicating a U-shaped relationship between BUN levels and all-cause mortality in individuals diagnosed with hyperlipidemia. The specific results indicate that in patients with hyperlipidemia, the all-cause mortality rates at BUN levels of less than 3.6 mmol/L and more than 6.09 mmol/L increased by 26% and 22%, respectively, compared to the control group. Concerning the impact of BUN on CVD mortality, these findings reveal a linear association between elevated BUN levels (> 6.09 mmol/L) and CVD mortality, consistent with prior NHANES data on the general population.

The production of BUN is directly influenced by daily protein intake [31]. Similarly, this investigation identified a positive relationship between BUN concentrations and protein intake. The liver plays a crucial role in the urea cycle, and liver damage can negatively impact urea nitrogen synthesis through various mechanisms [4, 32]. Moreover, elevated BUN levels are commonly observed in patients with renal insufficiency and are also observed in individuals with heart failure [33]. Nevertheless, after excluding individuals with baseline pregnancy and adjusting for protein intake, ALT, AST, eGFR, CVD, and lipid-lowering medications, a notable correlation between low and high levels of BUN and all-cause mortality remained. In stratified analyses, the study revealed a notable interaction between alcohol intake and hyperlipidemia. The impact of BUN was particularly prominent among hyperlipidemic patients who engaged in excessive alcohol consumpion. Therefore, regulating alcohol consumption in hyperlipidemic patients could potentially mitigate the risk of mortality. In addition, the impact of high levels of BUN on all-cause mortality was greater in patients taking lipid-lowering medications. This may be explained by the fact that patients taking lipid-lowering medications had a higher percentage of hypertension, diabetes mellitus, and CVD. Of note, the correlation between BUN concentrations and CVD mortality was no longer statistically significant after excluding participants with pre-existing CVD at baseline. This suggests that the impact of BUN on CVD in hyperlipidemia patients may be influenced by other confounding factors.

The mechanism of action of BUN in hyperlipidemia remains unclear at present. Previous research has reported that high urea levels can directly promote the progression of atherosclerosis by regulating the function and expression of pro-apoptotic proteins and inhibiting the proliferation of microvascular endothelial cells [34–36]. Moreover, urea serves as a source of cyanate and carbamoylated compounds [37]. Cyanate has been found to contribute to oxidative stress-induced liver damage and disrupt normal lipid metabolism [38]. Similarly, carbamoylated low-density lipoprotein uncouples endothelial nitric oxide synthase, directly contributing to endothelial dysfunction. Additionally, it can promote cholesterol accumulation and pro-inflammatory signaling by binding to macrophage scavenger receptors [39, 40].

### Strengths and limitations

The research conducted exhibits several strengths. This study assessed the correlation between BUN and mortality in individuals with hyperlipidemia utilizing a highly representative sample with a high-follow-up rate. Moreover, potential confounding variables such as lifestyle, dietary habits, and the use of lipid-lowering medications were also considered. However, it is critical to recognize certain limitations. Firstly, the study is observational, so the ability to infer causality is limited. Secondly, BUN concentrations were measured only once at baseline, which may limit their accuracy in reflecting long-term conditions. Thirdly, despite accounting for numerous covariates, it remains challenging to completely rule out residual or unidentified confounding factors.

## Conclusion

In this study, which utilized a sample representative of American adults with hyperlipidemia, a U-shaped correlation was observed between BUN levels and all-cause mortality, as well as a linear correlation with CVD mortality. Therefore, based on these findings, it is possible to manage the prognosis of patients with hyperlipidemia according to the concentration of BUN and to facilitate the development of individualized treatment plans.

### Data availability

The [NHANES] website [https://www.cdc.gov/nchs/nhanes/index.htm] provides free access to the data from the study.

### Electronic supplementary material

Below is the link to the electronic supplementary material.

Supplementary Material 1

Supplementary Material 2

Supplementary Material 3

## Data Availability

No datasets were generated or analysed during the current study.

## References

[1] Arvanitis M, Lowenstein CJ, Dyslipidemia (2023). Ann Intern Med.

[2] Tsao CW, Aday AW, Almarzooq ZI, Anderson CAM, Arora P, Avery CL (2023). Heart Disease and Stroke Statistics-2023 update: a Report from the American Heart Association. Circulation.

[3] Townsend N, Kazakiewicz D, Lucy Wright F, Timmis A, Huculeci R, Torbica A (2022). Epidemiology of cardiovascular disease in Europe. Nat Rev Cardiol.

[4] Higgins C. Urea and the clinical value of measuring blood urea concentration. Acutecaretesting Org. 2016:1–6.

[5] Wang H, Ran J, Jiang T, Urea (2014). Subcell Biochem.

[6] Peng R, Liu K, Li W, Yuan Y, Niu R, Zhou L (2021). Blood urea nitrogen, blood urea nitrogen to creatinine ratio and incident stroke: the Dongfeng-Tongji cohort. Atherosclerosis.

[7] Xie Y, Bowe B, Li T, Xian H, Yan Y, Al-Aly Z (2018). Higher blood urea nitrogen is associated with increased risk of incident diabetes mellitus. Kidney Int.

[8] Lin H, Wong GL, Zhang X, Yip TC, Liu K, Tse YK (2022). U-shaped relationship between urea level and hepatic decompensation in chronic liver diseases. Clin Mol Hepatol.

[9] Samuel VT, Petersen KF, Shulman GI (2010). Lipid-induced insulin resistance: unravelling the mechanism. Lancet.

[10] Miller YI, Shyy JY (2017). Context-dependent role of oxidized lipids and lipoproteins in inflammation. Trends Endocrinol Metab.

[11] Vanholder R, Gryp T, Glorieux G (2018). Urea and chronic kidney disease: the comeback of the century? (in uraemia research). Nephrol Dial Transpl.

[12] About the National health. and nutrition examination survey [Internet]. [cited 2023 October 23]. [https://www.cdc.gov/nchs/nhanes/about_nhanes.htm.

[13] the National Center for Health Statistics [Internet]. [cited 2023 October 23]. [https://www.cdc.gov/nchs/nhanes/index.htm.

[14] Detection, NCEPEPo. Adults ToHBCi: third report of the National Cholesterol Education Program (NCEP) Expert Panel on detection, evaluation, and treatment of high blood cholesterol in adults (Adult Treatment Panel III). The Program; 2002. 12485966

[15] NHANES 1999–2000. Laboratory Methods [Internet]. [cited 2023 October 23]. [https://wwwn.cdc.gov/nchs/nhanes/ContinuousNhanes/LabMethods.aspx?BeginYear=1999].

[16] NHANES 2001–2002. Laboratory Methods [Internet]. [cited 2023 October 23]. [https://wwwn.cdc.gov/nchs/nhanes/continuousnhanes/labmethods.aspx?Cycle=2001-2002].

[17] NHANES 2009–2010. Laboratory Methods [Internet]. [cited 2023 October 23]. [https://wwwn.cdc.gov/nchs/nhanes/continuousnhanes/labmethods.aspx?Cycle=2009-2010].

[18] NHANES 2017–2018. Laboratory Methods. [https://wwwn.cdc.gov/nchs/nhanes/continuousnhanes/labmethods.aspx?Cycle=2017-2018].

[19] 2019 Public-Use. Linked Mortality Files [Internet]. [cited 2023 October 23]. [https://www.cdc.gov/nchs/data-linkage/mortality-public.htm.

[20] Wan Z, Guo J, Pan A, Chen C, Liu L, Liu G (2021). Association of serum 25-Hydroxyvitamin D concentrations with all-cause and cause-specific mortality among individuals with diabetes. Diabetes Care.

[21] Li B, Chen L, Hu X, Tan T, Yang J, Bao W (2023). Association of serum uric acid with all-cause and Cardiovascular Mortality in Diabetes. Diabetes Care.

[22] Huang L, Lu Z, You X, Zou C, He L, Xie J (2023). U-shaped association of serum uric acid with all-cause mortality in patients with hyperlipidemia in the United States: a cohort study. Front Cardiovasc Med.

[23] Shen S, Yan X, Xu B (2022). The blood urea nitrogen/creatinine (BUN/cre) ratio was U-shaped associated with all-cause mortality in general population. Ren Fail.

[24] Liu Y, Geng T, Wan Z, Lu Q, Zhang X, Qiu Z (2022). Associations of serum folate and vitamin B12 levels with Cardiovascular Disease Mortality among patients with type 2 diabetes. JAMA Netw Open.

[25] Whelton PK, Carey RM, Aronow WS, Jr DE Casey, Collins KJ, Dennison Himmelfarb C (2018). 2017 ACC/AHA/AAPA/ABC/ACPM/AGS/APhA/ASH/ASPC/NMA/PCNA Guideline for the Prevention, detection, evaluation, and management of high blood pressure in adults: a report of the American College of Cardiology/American Heart Association Task Force on Clinical Practice guidelines. J Am Coll Cardiol.

[26] ElSayed NA, Aleppo G, Aroda VR, Bannuru RR, Brown FM, Bruemmer D (2023). 2. Classification and diagnosis of diabetes: standards of Care in Diabetes-2023. Diabetes Care.

[27] Levey AS, Stevens LA, Schmid CH, Zhang Y, Castro AF, Feldman HI (2009). A new equation to estimate glomerular filtration rate. Ann Intern Med.

[28] Hong C, Zhu H, Zhou X, Zhai X, Li S, Ma W (2023). Association of Blood Urea Nitrogen with Cardiovascular diseases and all-cause mortality in USA adults: results from NHANES 1999–2006. Nutrients.

[29] Lind L, Zanetti D, Hogman M, Sundman L, Ingelsson E (2020). Commonly used clinical chemistry tests as mortality predictors: results from two large cohort studies. PLoS ONE.

[30] Seki M, Nakayama M, Sakoh T, Yoshitomi R, Fukui A, Katafuchi E (2019). Blood urea nitrogen is independently associated with renal outcomes in Japanese patients with stage 3–5 chronic kidney disease: a prospective observational study. BMC Nephrol.

[31] Dinu M, Colombini B, Pagliai G, Giangrandi I, Cesari F, Gori A (2021). Effects of vegetarian versus Mediterranean diet on kidney function: findings from the CARDIVEG study. Eur J Clin Invest.

[32] Glavind E, Aagaard NK, Gronbaek H, Moller HJ, Orntoft NW, Vilstrup H (2016). Alcoholic Hepatitis markedly decreases the capacity for Urea Synthesis. PLoS ONE.

[33] Richter B, Sulzgruber P, Koller L, Steininger M, El-Hamid F, Rothgerber DJ (2019). Blood urea nitrogen has additive value beyond estimated glomerular filtration rate for prediction of long-term mortality in patients with acute myocardial infarction. Eur J Intern Med.

[34] Trécherel E, Godin C, Louandre C, Benchitrit J, Poirot S, Mazière J-C (2012). Upregulation of BAD, a pro-apoptotic protein of the BCL2 family, in vascular smooth muscle cells exposed to uremic conditions. Biochem Biophys Res Commun.

[35] D’Apolito M, Du X, Pisanelli D, Pettoello-Mantovani M, Campanozzi A, Giacco F (2015). Urea-induced ROS cause endothelial dysfunction in chronic renal failure. Atherosclerosis.

[36] Colombo G, Altomare A, Astori E, Landoni L, Garavaglia ML, Rossi R (2022). Effects of physiological and pathological urea concentrations on human microvascular endothelial cells. Int J Mol Sci.

[37] Badar A, Arif Z, Alam K (2018). Role of Carbamylated Biomolecules in Human diseases. IUBMB Life.

[38] Hu L, Tian K, Zhang T, Fan CH, Zhou P, Zeng D (2019). Cyanate Induces Oxidative Stress Injury and abnormal lipid metabolism in liver through Nrf2/HO-1. Molecules.

[39] Wang Z, Nicholls SJ, Rodriguez ER, Kummu O, Hörkkö S, Barnard J (2007). Protein carbamylation links inflammation, smoking, uremia and atherogenesis. Nat Med.

[40] Speer T, Owala FO, Holy EW, Zewinger S, Frenzel FL, Stähli BE (2014). Carbamylated low-density lipoprotein induces endothelial dysfunction. Eur Heart J.

