# Supplementary material for: Association of blood urea nitrogen with all-cause and cardiovascular mortality in hyperlipidemia: NHANES 1999–2018
Source: Lipids Health Dis. 2024 Jun 3;23:164. doi: 10.1186/s12944-024-02158-1 (PMC11145831; doi:10.1186/s12944-024-02158-1)
Supplement: Supplementary file 1 — Supplementary Material 1 [file 12944_2024_2158_MOESM1_ESM.docx]

**Supplementary Table 1. Subgroup analysis of the association between BUN levels and CVD mortality in patients with hyperlipidemia.**

|  | **BUN, mmol/L** | | | | |  |
| --- | --- | --- | --- | --- | --- | --- |
| **Subgroups** | **Q1 <3.60** | **Q2 3.60-4.29** | **Q3 4.30-5.00** | **Q4 5.01-6.09** | **Q5 >6.09** | **P interaction** |
| **Age, years** |  |  |  |  |  | 0.283 |
| <60 | 0.80 (0.46, 1.38) | 1.34 (0.71, 2.54) | 1(ref) | 1.44 (0.73, 2.83) | 1.84 (0.90, 3.78) |  |
| ≥60 | 0.99 (0.68, 1.43) | 1.10 (0.77, 1.58) | 1(ref) | 1.24 (0.92, 1.68) | 1.47 (1.13, 1.91) |  |
| **Sex** |  |  |  |  |  | 0.916 |
| Female | 1.14 (0.73, 1.77) | 1.35 (0.90, 2.02) | 1(ref) | 1.39 (0.90, 2.10) | 1.72 (1.17, 2.52) |  |
| Male | 1.01 (0.68, 1.50) | 1.21 (0.74, 1.97) | 1(ref) | 1.18 (0.82, 1.70) | 1.36 (0.96, 1.93) |  |
| **Race/ethnicity** |  |  |  |  |  | 0.723 |
| Non-Hispanic White | 1.05 (0.69, 1.58) | 1.12 (0.75, 1.66) | 1(ref) | 1.21 (0.87, 1.68) | 1.43 (1.04, 1.96) |  |
| Other | 1.02 (0.65, 1.61) | 1.62 (1.02, 2.56) | 1(ref) | 1.41 (0.90, 2.21) | 1.59 (1.05, 2.39) |  |
| **Education level** |  |  |  |  |  | 0.558 |
| Less than high school | 1.14 (0.74, 1.77) | 1.02 (0.61, 1.70) | 1(ref) | 1.36 (0.88, 2.09) | 1.62 (1.14, 2.31) |  |
| High school | 0.70 (0.36, 1.36) | 1.24 (0.69, 2.24) | 1(ref) | 1.04 (0.64, 1.67) | 1.21 (0.82, 1.79) |  |
| College or higher | 1.21 (0.72, 2.03) | 1.33 (0.83, 2.14) | 1(ref) | 1.42 (0.96, 2.09) | 1.57 (1.00, 2.44) |  |
| **PIR** |  |  |  |  |  | 0.304 |
| ≤1.30 | 1.36 (0.88, 2.11) | 1.35 (0.82, 2.21) | 1(ref) | 1.52 (0.98, 2.34) | 1.56 (0.94, 2.58) |  |
| 1.31-3.50 | 0.83 (0.49, 1.40) | 0.95 (0.59, 1.52) | 1(ref) | 0.97 (0.67, 1.40) | 1.15 (0.83, 1.58) |  |
| >3.50 | 0.78 (0.38, 1.61) | 1.65 (0.84, 3.26) | 1(ref) | 1.58 (0.90, 2.79) | 2.06 (1.17, 3.62) |  |
| **BMI, kg/m^2^** |  |  |  |  |  | 0.706 |
| <25.0 | 0.99 (0.59, 1.66) | 1.05 (0.57, 1.93) | 1(ref) | 1.55 (0.98, 2.46) | 1.29 (0.87, 1.92) |  |
| 25.0-29.9 | 0.89 (0.50, 1.58) | 1.24 (0.74, 2.09) | 1(ref) | 1.22 (0.77, 1.93) | 1.56 (1.01, 2.40) |  |
| ≥30.0 | 1.22 (0.74, 1.99) | 1.37 (0.85, 2.20) | 1(ref) | 1.17 (0.76, 1.80) | 1.54 (1.05, 2.27) |  |
| **Smoking status** |  |  |  |  |  | 0.898 |
| Never | 1.06 (0.67, 1.68) | 1.51 (1.04, 2.19) | 1(ref) | 1.30 (0.96, 1.76) | 1.48 (1.08, 2.04) |  |
| Former | 0.90 (0.52, 1.57) | 1.28 (0.77, 2.13) | 1(ref) | 1.36 (0.93, 2.00) | 1.47 (1.01, 2.13) |  |
| Current | 0.93 (0.55, 1.57) | 0.89 (0.43, 1.83) | 1(ref) | 1.10 (0.56, 2.13) | 1.39 (0.75, 2.55) |  |
| **Alcohol intake** |  |  |  |  |  | 0.272 |
| None | 1.04 (0.71, 1.54) | 1.21 (0.86, 1.70) | 1(ref) | 1.40 (1.08, 1.82) | 1.56 (1.19, 2.04) |  |
| Moderate drinking | 0.82 (0.37, 1.79) | 1.88 (0.95, 3.71) | 1(ref) | 0.84 (0.42, 1.71) | 1.04 (0.61, 1.77) |  |
| Heavy drinking | 0.93 (0.33, 2.61) | 0.78 (0.23, 2.64) | 1(ref) | 0.73 (0.26, 2.04) | 1.70 (0.75, 3.87) |  |
| **Physical activity** |  |  |  |  |  | 0.660 |
| Inactive | 1.05 (0.72, 1.53) | 1.06 (0.75, 1.49) | 1(ref) | 1.15 (0.83, 1.60) | 1.28 (0.95, 1.72) |  |
| Active | 0.98 (0.55, 1.74) | 1.54 (0.91, 2.61) | 1(ref) | 1.35 (0.88, 2.07) | 1.74 (1.17, 2.57) |  |
| **Diabetes** |  |  |  |  |  | 0.936 |
| Yes | 0.72 (0.39, 1.35) | 1.00 (0.63, 1.59) | 1(ref) | 1.20 (0.74, 1.94) | 1.55 (0.94, 2.55) |  |
| No | 1.19 (0.86, 1.65) | 1.35 (0.92, 1.96) | 1(ref) | 1.30 (0.95, 1.79) | 1.42 (1.05, 1.92) |  |
| **Hypertension** |  |  |  |  |  | 0.420 |
| Yes | 0.97 (0.69, 1.38) | 1.29 (0.93, 1.79) | 1(ref) | 1.15 (0.90, 1.48) | 1.50 (1.13, 2.00) |  |
| No | 1.29 (0.59, 2.81) | 1.09 (0.50, 2.38) | 1(ref) | 1.68 (0.85, 3.33) | 1.43 (0.75, 2.72) |  |
| **CVD** |  |  |  |  |  | 0.402 |
| Yes | 1.14 (0.69, 1.88) | 0.97 (0.56, 1.69) | 1(ref) | 1.42 (0.90, 2.23) | 1.53 (1.00, 2.35) |  |
| No | 1.00 (0.67, 1.47) | 1.42 (0.99, 2.04) | 1(ref) | 1.19 (0.89, 1.58) | 1.49 (1.10, 2.02) |  |
| **Medications** |  |  |  |  |  |  |
| Yes | 1.06 (0.74, 1.53) | 0.98 (0.67, 1.41) | 1(ref) | 1.26 (0.94, 1.69) | 1.49 (1.13, 1.96) | 0.128 |
| No | 1.15 (0.68, 1.95) | 1.85 (1.10, 3.11) | 1(ref) | 1.30 (0.77, 2.19) | 1.47 (0.85, 2.55) |  |
| **eGFR, mL/min/1.73m^2^** |  |  |  |  |  |  |
| <90 | 1.04 (0.65, 1.65) | 1.31 (0.80, 2.12) | 1(ref) | 1.61 (1.17, 2.23) | 2.04 (1.49, 2.79) | 0.807 |
| ≥90 | 1.02 (0.67, 1.56) | 1.24 (0.83, 1.86) | 1(ref) | 1.19 (0.78, 1.80) | 1.43 (0.90, 2.29) |  |
| Values are weighted hazard ratio (95% confidence interval). Models adjusted for age, sex, race/ethnicity, education level,PIR, BMI, smoking status, alcohol intake, protein intake, physical activity, diabetes, hypertension, CVD, medications, eGFR, Albumin, ALT, AST, serum uric acid, excluding the stratifying variable. | | | | | | |
