# Supplementary material for: Association of blood urea nitrogen with all-cause and cardiovascular mortality in hyperlipidemia: NHANES 1999–2018
Source: Lipids Health Dis. 2024 Jun 3;23:164. doi: 10.1186/s12944-024-02158-1 (PMC11145831; doi:10.1186/s12944-024-02158-1)
Supplement: Supplementary file 2 — Supplementary Material 2 [file 12944_2024_2158_MOESM2_ESM.docx]

**Supplementary Table 2. Association of blood urea nitrogen with all-cause and CVD mortality in patients with hyperlipidemia in NHANES 1999-2018 after excluding the died within twe years.**

|  | **BUN, mmol/L** | | | | |  |
| --- | --- | --- | --- | --- | --- | --- |
|  | **Q1 <3.57** | **Q2 3.57-4.29** | **Q3 4.30-5.00** | **Q4 5.01-6.09** | **Q5 >6.09** | **P trend** |
| **All-cause mortality** |  |  |  |  |  |  |
| Model1 | 1.68 (1.45, 1.95) | 1.10 (0.95, 1.27) | 1(ref) | 1.04 (0.91, 1.19) | 1.41 (1.26, 1.59) | <0.001 |
| Model2 | 1.36 (1.18 1.56) | 1.04 (0.89, 1.21) | 1(ref) | 1.08 (0.94, 1.23) | 1.41 (1.25, 1.60) | <0.001 |
| Model3 | 1.33 (1.14, 1.54) | 1.01 (0.87, 1.18) | 1(ref) | 0.99 (0.86, 1.14) | 1.17 (1.05, 1.32) | <0.001 |
| **CVD mortality** |  |  |  |  |  |  |
| Model1 | 1.43 (0.95, 2.14) | 1.17 (0.83, 1.65) | 1(ref) | 1.34 (1.02, 1.77) | 1.94 (1.50, 2.50) | <0.001 |
| Model2 | 1.15 (0.77, 1.72) | 1.11 (0.79, 1.56) | 1(ref) | 1.39 (1.05, 1.85) | 1.91 (1.46, 2.49) | <0.001 |
| Model3 | 1.14 (0.76, 1.69) | 1.09 (0.78, 1.51) | 1(ref) | 1.25 (0.94, 1.66) | 1.47 (1.12, 1.92) | 0.051 |
| HR (95% CI) was estimated by weighted Cox regression analysis. Model 1: adjusted for age, sex, race/ethnicity. Model 2: Model 1 + education, PIR, smoking status, alcohol intake, protein intake, physical activity, BMI. Model 3: Model 2 + diabetes, hypertension, CVD, medications, eGFR, albumin, ALT, AST, serum uric acid. | | | | | | |
