# Supplementary material for: Association of blood urea nitrogen with all-cause and cardiovascular mortality in hyperlipidemia: NHANES 1999–2018
Source: Lipids Health Dis. 2024 Jun 3;23:164. doi: 10.1186/s12944-024-02158-1 (PMC11145831; doi:10.1186/s12944-024-02158-1)
Supplement: Supplementary file 3 — Supplementary Material 3 [file 12944_2024_2158_MOESM3_ESM.docx]

**Supplementary Table 3. Association of blood urea nitrogen with all-cause and CVD mortality in patients with hyperlipidemia in NHANES 1999-2018 after excluding the patients with a history of CVD at baseline**

|  | **BUN, mmol/L** | | | | |  |
| --- | --- | --- | --- | --- | --- | --- |
|  | **Q1 <3.57** | **Q2 3.57-4.29** | **Q3 4.30-5.00** | **Q4 5.01-6.09** | **Q5 >6.09** | **P trend** |
| **All-cause mortality** |  |  |  |  |  |  |
| Model1 | 1.82 (1.55, 2.14) | 1.15 (0.99, 1.34) | 1(ref) | 1.00 (0.85, 1.17) | 1.33 (1.15, 1.53) | <0.001 |
| Model2 | 1.42 (1.24, 1.69) | 1.09 (0.94, 1.28) | 1(ref) | 1.04 (0.89, 1.21) | 1.34 (1.16, 1.56) | <0.001 |
| Model3 | 1.43 (1.20, 1.70) | 1.09 (0.93, 1.28) | 1(ref) | 0.99 (0.84, 1.17) | 1.21 (1.05, 1.39) | <0.001 |
| **CVD mortality** |  |  |  |  |  |  |
| Model1 | 1.42 (0.92, 2.17) | 1.25 (0.91, 1.72) | 1(ref) | 1.23 (0.92, 1.65) | 1.77 (1.33, 2.34) | 0.001 |
| Model2 | 1.14 (0.74, 1.76) | 1.20 (0.86, 1.66) | 1(ref) | 1.28 (0.96, 1.70) | 1.74 (1.30, 2.33) | 0.003 |
| Model3 | 1.17 (0.76, 1.80) | 1.21 (0.87, 1.69) | 1(ref) | 1.18 (0.89, 1.58) | 1.48 (1.09, 2.01) | 0.164 |
| HR (95% CI) was estimated by weighted Cox regression analysis. Model 1: adjusted for age, sex, race/ethnicity. Model 2: Model 1 + education, PIR, smoking status, alcohol intake, protein intake, physical activity, BMI. Model 3: Model 2 + diabetes, hypertension, CVD, medications, eGFR, albumin, ALT, AST, serum uric acid. | | | | | | |
